# Knowledge, perceptions and experiences of risk to sexual violence among adults with intellectual disabilities in Cape Town, South Africa

**DOI:** 10.4102/ajod.v11i0.837

**Published:** 2022-03-18

**Authors:** Callista K. Kahonde, Rebecca Johns

**Affiliations:** 1 Department of Global Health, Centre for Disability and Rehabilitation Studies, Faculty of Medicine and Health Sciences, Stellenbosch University, Cape Town, South Africa; 2 Consultant for Western Cape Forum for Intellectual Disability, Cape Town, South Africa

**Keywords:** intellectual disabilities, sexual violence, abuse, risk, knowledge, perceptions, experiences

## Abstract

**Background:**

People with intellectual disabilities are at high risk to sexual violence, yet minimal research has been conducted in South Africa to understand this phenomenon, especially seeking perspectives of people with intellectual disabilities themselves.

**Objectives:**

This study aimed to explore and describe the knowledge and awareness of risk to sexual violence among adults with intellectual disabilities and to understand their perceptions and experiences of risk.

**Method:**

An exploratory qualitative approach was appropriate as there is lack of literature on this subject. Focus group discussions were used as the method of data collection. The method of conducting focus group discussions and data collection instruments were adapted to suit the communication and cognitive abilities of the adults. Twenty-seven adults participated in the study and they were divided into six groups of four to five participants in each group.

**Results:**

The adults’ responses revealed that they had some knowledge of risks to sexual violence, but they also had knowledge gaps and some erroneous knowledge and perceptions that could put them at high risk. The experiences they shared showed that the risk of sexual violence is high among women with intellectual disabilities.

**Conclusion:**

Further research is needed to inform a community approach which includes people with intellectual disabilities, their families, services providers and community members as an intervention to empower and protect people with intellectual disabilities from sexual violence. To achieve this, we recommend an ecological framework as a guiding tool in both the research processes and the implementation of the outcomes.

## Introduction

Sexual violence is rife in South Africa. The World Population Review reported that South Africa has the highest incidents of rape in the world, with an alarming rate of 132.4 incidents per 100 000 people (World Population Review 2020). There is a lack of disaggregated data presenting the statistics of sexual offences against people with intellectual disabilities in this country. However, the evidence of their high vulnerability worldwide and the scanty local literature available suggests that this group is particularly at high risk (Dickman & Roux 2005; Meer & Combrinck 2015).

Research specifically seeking to understand sexual violence against people with intellectual disabilities in South Africa is limited. The few areas that have been addressed on this subject mostly focus on sexual violence among learners with intellectual disabilities from the school setting (Mdikana, Phasha & Ntshangase 2018; Nyokangi & Phasha 2016; Phasha 2009, 2013; Phasha & Myaka 2014; Phasha & Nyokangi 2012). Sexual violence has also been studied in research exploring gender-based violence among women with intellectual disabilities (Meer & Combrinck 2015). Of the school-based studies, only Phasha and Nyokangi (2012) and Nyokangi and Phasha (2016) sought the experiences of learners with intellectual disabilities and the rest interviewed professionals, families or community members as their participants.

The scanty literature highlighting the voice of people with intellectual disabilities raise the need to hear their own perceptions and experiences of sexual violence so as to identify their gaps in knowledge and awareness of risks. Against this backdrop, the current study explored the knowledge, experiences and perceptions of risk to sexual violence among adults with mild to moderate intellectual disabilities receiving services at facilities in Cape Town, South Africa. We see this as a critical step towards a *nothing about us without us* approach in research seeking to understand sexual violence among people with intellectual disabilities. Previous research that mostly relied on responses from proxies was critiqued by Hollomotz (2018), because the proxy is likely to express their own experiences and subjective perceptions and not necessarily represent the person concerned. The practice of relying on proxies reflects society’s belief that people with intellectual disabilities are not able to speak for themselves. This view sees adults with intellectual disabilities as perpetual children and it impacts on their confidence and may intensify their vulnerability to all forms of abuse (Nyokangi & Phasha 2016). On the contrary, giving them space to speak for themselves while affirming their positive contributions and assisting them to recognise erroneous perceptions as enabled by the methodology of this study is both empowering and emancipating for people with intellectual disabilities.

This study is the first of its kind in the study setting. The purpose was to explore and describe the knowledge, perceptions and experiences of risk to sexual violence among adults with intellectual disabilities with the aim to identify gaps and opportunities to inform services for their empowerment and prevention of sexual violence for this group. As a starting point, an exploratory approach was appropriate as there is lack of literature on this subject. It is expected that the study findings will give impetus to more in-depth, theory informed studies in the future. A relevant theoretical lens suggested by the study findings, which will be expounded on later is the Ecological Systems Theory (Bronfenbrenner 1979).

## Literature review

Although the trends may vary by context, sexual violence against people with intellectual disabilities is an issue of concern in both high income and low to middle income countries among children and adults with this type of disability (Curtiss & Kammes 2020; Lin et al. 2009; Smeaton & Franklin 2018). In Taiwan, Lin et al. (2009) reported that sexual assault among people with intellectual disabilities comprised more than 50% of the statistics of sexual assault across all types of disabilities. In the UK, Smeaton and Franklin (2018) found that children with intellectual disabilities are more vulnerable to child sexual abuse when compared to their counterparts without disabilities. In a more recent study, Majeed-Ariss, Rodriguez and White (2020) reported an over-representation of people with intellectual disabilities among people seeking forensic medical examination at Saint Marys Sexual Assault Referral Centre. In the USA, Shapiro (2018) presented a special series of articles on National Public Radio that explored sexual violence against people with intellectual disabilities reporting unpublished data from the US Justice Department. The data showed that people with intellectual disabilities were assaulted at rates more than seven times higher than the population of people without disabilities. Research on the prevalence of sexual violence against people with intellectual disabilities in African countries other than South Africa is lacking, but there is also evidence of higher occurrences among this group. For example, in Nigeria, Aderemi and Pillay (2013) found that the rate of sexual abuse was four times higher for school-going adolescent girls with mild to moderate intellectual disabilities when compared to the adolescents without disabilities who were the control group in their study.

The reasons for increased vulnerability of people with intellectual disabilities documented in the literature are related to their limitations in intellectual and adaptive functioning, and negative attitudes and perceptions towards their sexuality (Phasha & Myaka 2014; Smeaton & Franklin 2018). It is important to understand both the contextual risks and the impairment-related risks so that as a society we can work towards taking action against this scourge without simply blaming it on the attributes of the individuals with intellectual disabilities. In line with this, Curtiss and Kammes (2020) argued that individual factors on their own do not cause sexual violence; hence, the interplay between the impairment and the environment should be considered. Phasha and Myaka (2014), in their study on factors contributing to the vulnerability of teenagers with intellectual disabilities to sexual abuse found an interaction of individual, family and community factors as central in putting the teenagers at risk. They argued for preventative interventions that take consideration of what happens at all the three levels. Such interventions require the understanding of perceptions and experiences of people with intellectual disabilities and not just relying on research documenting the perceptions of carers, professionals, and others.

People with intellectual disabilities are unlikely to receive adequate sexuality education because of misconceptions and myths, for example, the belief that they are asexual or hypersexual (Shakespeare 2013) and the lack of understanding of how much they can comprehend (Aderemi & Pillay 2013). There is also a common fear, especially among family caregivers that sexuality education may ‘wake sleeping dogs’ by awakening an interest in sexual activity (Kahonde 2016). Myths, stereotypes and negative attitudes towards intellectual disability have been found to heighten their risk to sexual abuse within the South African context (Meer & Combrinck 2015; Phasha & Myaka 2014). Phasha and Myaka (2014) reported that professionals and community members in their study in Gauteng province believed that learners with intellectual disabilities have a high sex drive or they are naturally sexually attractive because of ‘spirits’ and ‘powers’ related to intellectual disabilities. The myths and misconceptions and the factors that put people with intellectual disabilities at higher risk need to be addressed through evidence-informed interventions. There is also a gender bias within research focusing on sexual violence against people with intellectual disabilities. Most studies focus on women with intellectual disabilities (Barger et al. 2009; Bernet & Ogletree 2013; Bornman & Rathbone 2016; Haffejee & Theron 2017; Meer & Combrink 2015). Very little is known about the sexual violence against men with intellectual disabilities. Hence, this study attempted to understand this phenomenon from the perspectives of male and female adults with intellectual disabilities.

## Methods

### Study setting and participants

The study was conducted in Cape Town, South Africa at facilities offering services to adults with intellectual disabilities. It was part of a bigger study that explored the risk of sexual violence against people with intellectual disabilities with the ultimate aim of developing innovative ways to support empowerment and to decrease risk. Twenty-seven adults with mild to moderate intellectual disabilities between the age of 20 and 47 years participated in the study. There were 18 women and 9 men. The participants had to be able to communicate verbally to be eligible to participate. The level of intellectual disability was not assessed for the purposes of the study but was determined as classified by the service providers. The demographic information of the participants is shown in Table 1.

**TABLE 1:** Details of the groups and participants.

Focus group	Pseudonyms	Number of sessions attended	Gender	Language	Presence of support staff
FG1	Lidia	4	Female	Afrikaans	Yes
	Prisca	4			
	Angela	4			
	Prim	2			
FG2	Mary	4	Female	Afrikaans/English	Yes
	Molly	4			
	Leah	3			
	Nicole	4			
FG3	Deon	4	Female	Afrikaans	Yes
	Thato	4			
	Talent	4			
	Zoe	2			
	Evelyn	3			
FG4	Joe	4	Male	Afrikaans	Yes
	Dylan	3			
	Tom	4			
	Ethan	3			
FG5	Mavis	3	Female	English	No
	Betty	3			
	Ella	4			
	Agnes	2			
	Emily	4			
FG6	Victor	4	Male	English/IsiXhosa	Yes
	Luke	4			
	Nathan	3			
	Abel	3			
	Jabu	3			

FG, focus group.

### Recruitment

The participants were recruited through facilities offering services for adults with intellectual disabilities. The participants were purposively selected with the help of the coordinators and managers of the facilities to include participants from different backgrounds in terms of language, ethnicity and socio-economic status. The principal investigator wrote letters seeking permission to the family caregivers which the coordinators of the facilities gave the adults with intellectual disabilities to deliver to their families with a reply slip. Before the study commenced, the workshop coordinators or another service provider working with the adults played an intermediate and supportive role between the researchers and the participants by explaining what the letters were saying and also describing what was expected of the adults. During this process, the service providers emphasised that the participation was voluntary and the adults had the right to choose to participate in the study or not even if their parents gave consent for them to participate.

Only the adults whose family caregivers gave consent for them to participate were included in the study as the rules of the facilities did not allow them to participate without consent from the family. This was a limitation to the recruitment process which the researchers had no control of. This gatekeeping around the participation of people with intellectual disabilities in research is one of the possible reasons for the lack of research directly documenting the voices of people with intellectual disabilities. Carlson (2013:305) calls this the ‘double danger of inclusion or exclusion’; whereby on the one hand people with intellectual disabilities are viewed as a vulnerable group in need of special consideration and protection in relation to research while on the other hand these special considerations may result in their exclusion from research and their direct experience and perspective remaining invisible.

### Data collection

Data were collected using focus group interviews. Twenty-four focus group discussions were conducted with six groups of adults with intellectual disabilities. A series of four sessions were held with each group, and the sessions were conducted weekly for each group with each session theme building on the previous one. Each group session lasted for an hour. Not all participants were able to attend the four sessions. Some of them missed one or two sessions because of illness, and work or family commitments but there were at least three participants at each group session. There were four groups of female participants and two groups of male participants. The groups were separated according to gender because of the sensitive and potentially triggering nature of the subject which might have made it more difficult for participants to feel safe in a mixed group. The group allocation was performed by the staff at the facilities and none of the participants presented or disclosed as identified as lesbian, gay, bisexual, transgender, queer, intersex, asexual, pansexual (LGBTQIAP). However, the focus group discussions included aspects of different types of relationships and also allowed the participants to choose the orientation of characters in relationships enabling activities and data collection tools to transcend depictions of solely heterosexual relationships.

Four groups mixed Afrikaans and English, one group mixed IsiXhosa and English and one was conducted in English only. The staff members from the facilities (known to and supportive of the adults) assisted with translation in the groups with non-English speaking participants.

All the sessions were conducted in a comfortable, private room at the facility where the adults were residing or receiving day services. The first session started with the informed consent process and setting rules for the group discussions, followed by ice-breaking activities which helped the participants to relax. The activities aimed to build rapport between the participants and the researchers and for researchers to gain some understanding of the participants’ communication abilities. Sessions two to four focused on themes such as relationships and feelings, consent and seeking help and awareness of abuse. Concepts were introduced progressively, starting with less sensitive subjects like different types of emotions, relationships, boundaries, knowing your body, private and public, and then progressed to more sensitive subjects like intimacy, giving or refusing consent to sexual behaviour and considering strategies in response to the experience of sexual abuse.

The data collection process used tools to make the concepts simpler and concrete to the participants (Hollomotz 2018). The concrete tools included emojis, pictures of relationships, pictures of consenting and non-consenting touch and cut-out characters used to co-create social stories with the group. These picture resources form part of a sexuality education programme developed by the Western Cape Forum for Intellectual Disability (Johns 2020). Pictures and social stories are a recognised method to facilitate communication and learning with people with intellectual disabilities, particularly those with limited literacy (Bornman & Rathbone 2016). These tools allowed the group to share their views while depersonalising sensitive themes onto the pictures or characters. The participants were encouraged to interpret the relationship shown in the pictures through reading body language and/or emotions and whether the situation looks safe or unsafe, if the characters are both consenting and the steps they should take if they are not consenting. The group also co-created social stories using the cut-out characters that allowed them to safely share their perceptions of relationships, boundaries, and strategies to seek help and support where needed.

The researchers were attentive to ‘teaching moments’ during the focus group discussions whereby they would affirm the participants’ responses who showed an awareness of their rights, healthy relationships, risky situations and strategies for self-protection. The participants were provided with more information, guidance and/or correction when they suggested an inappropriate or erroneous response. Having four sessions afforded opportunities to strengthen the group’s awareness of their right to report, to seek help and not to be abused, as a way of providing some benefit for their participation and contribution to the study. Both authors were present during all the focus group discussions. Author 1 opened the sessions with an icebreaker followed by the informed consent process and the rules of conduct and observed and took notes throughout the sessions. Author 2 did most of the interview activities with the participants. The interviews were recorded with a voice recorder.

The researchers were both female. All the male groups and the female non-English speaking groups had a supporter of the same gender as the participant, who was a staff member at the facility. The facilitators attempted to put the male participants at ease by acknowledging that the participants might find it difficult to discuss sexuality issues with female researchers and they were reassured that the focus groups were a safe space and they were given the opportunity to decide rules to guide the group sessions. The ice-breaking activities at the beginning of each session helped the participants to relax.

### Data analysis

The two researchers met after every session to reflect and share their thoughts about the session. Details of the reflection were written as part of the notes from that specific session. The focus group interview recordings were transcribed verbatim and all the parts of the interviews which were in IsiXhosa or Afrikaans were translated to English. The verbatim transcripts were then analysed by Author 1 following the approach to thematic analysis by Braun and Clarke (2006, 2019). The initial phase, familiarisation with data, was started during the shared reflections between the two authors and was continued through reading and re-reading of the transcripts. This was followed by systematic coding of the data through naming of phrases, words and chunks of data from which meaning could be derived. Codes with similar traits were then grouped into initial themes and sub-themes. This was followed by further analysis of the initial themes and sub-themes and re-arranging of some of the codes within or across themes as appropriate to ensure coherence and fit. At this stage, the themes were shared with Author 2 for verification and the two authors collaborated in the final phase of defining and naming of themes.

### Ethical considerations

The study was approved by the Stellenbosch University, Faculty of Health Sciences Human Research Ethics Committee (reference number: N19/06/072). The informed consent process used a pictorial form with headings and very little text as most of the participants were unable to read but they could link pictures to the verbally explained concepts which supported understanding and retention of the information. The participants were given space to ask questions during and after going through the informed consent form and took the consent form home to show their family. They all signed their own forms by either writing their name, initials or putting an ‘X’ if they were unable to write. A code of conduct for focus group discussions was discussed and the participants were asked to add what was important to them in terms of how the group discussions were going to be conducted. All the subsequent sessions started with a recap of the informed consent and the code of conduct. Given the sensitive nature of the subject of inquiry, it was anticipated that some participants may be emotionally affected by the conversations, so the researchers made prior arrangements with the facilities for the social workers to be on stand-by to speak to anyone needing counselling. The informed consent process explained that if any participant disclosed a previous or current experience of being harmed or abused in the session, the researchers would need to tell someone like a social worker in their organisation so that they could receive the support they need, but that no communication would happen without their knowledge and involvement. The participants’ identities are protected by the use of pseudonyms in this article and all other documents reporting the study.

## Findings

The discussions with the 27 participants revealed that they had some knowledge of their rights and the risks to sexual violence, but they also had knowledge gaps and some erroneous knowledge and perceptions that could further increase their risk of abuse. Generally, women were more spontaneous and participative than the men who needed more probing and encouragement to join the discussions. The four themes that were generated from the data were as follows: ‘Experiences and perceptions of love relationships; Knowledge and awareness of sexual abuse; Past experience of abuse/attempted abuse and Knowing what to do in case of abuse’.

It is important to note that although the questions were presented using the concrete tools to depersonalise sensitive themes, there were some participants who prompted by a story scenario or picture, chose to share their personal experiences as shown by some of the excerpts below.

### Experiences and perceptions of love relationships

Discussions of what a healthy love relationship entails and seeing pictures of couples led to eight out of the 27 participants sharing that they were in love relationships. Only one of the male participants spoke about his girlfriend and the rest of those who spoke about their relationships were women. There was also one male participant who shared that he had a 2-year-old daughter but he was not together with the mother of the child. All the relationships were heterosexual and they involved a partner who also had intellectual disability. Most relationships seemed to be more platonic than sexual as they indicated that they were not keen on engaging in sexual touch or ‘going to bed together’, but preferred sitting together and cuddling. Some of their responses indicated that sexual intimacy was discouraged by parents and service providers. They had to say the following about their relationships:

‘It’s not nice…I don’t like when someone touches private stuff…in the bed…not my favourite…I love cuddle and stuff…that’s my favourite…and one kiss…that’s all….I don’t like French kissing….’ (Lidia, Female, 32, Afrikaans, FG1)

In agreement to what Lidia had said, Prisca interjected saying:

‘Yes it is different. It’s almost like X and I…on occasion we sometimes hold hands…or sometimes he will whisper something into my ear. He will always buy me chocolates…I like that….’ (Prisca, Female, 36, Afrikaans, FG1)

One was interested in having a boyfriend and probably a sexual relationship, but her mother was discouraging her:

‘I think I like to kiss. I don’t have a boyfriend…My mother says that I mustn’t get a boyfriend… the boyfriend want to kiss…and go ahead. Yesterday my mother says that the guys naked … then they tighten their hands around the girls hair … I don’t want to do that…my mother said that they are doing other things in their relationship….’ (Deon, Female, 31, Afrikaans, FG3)

One who had her boyfriend attending the same workshop with her shared that the manager told them that the men are not allowed to touch the women. The couples at the workshop were receiving regular counselling and guidance from the manager and being given rules on how to behave at the workshop:

‘We went to a meeting at the manager … and she said that the men are not allowed to touch us. We always have a couples meeting … and that’s why I keep it in my brain.’ (Thato, Female, 41, Afrikaans, FG3)

Abel, the man in Focus Group 3 who shared about his girlfriend said, ‘Y is my special friend. She helps me with things like doing stuff on my phone and reading my messages’. Although he had shared about this ‘special friend’, he later on became very withdrawn and looked upset during the focus group sessions. The researchers asked him if he wanted to continue and he said he wanted to continue, but he did not like talking about sex because his parents never talk about such things with him.

While others expressed lack of interest or disapproval of sexual touch, some responded to a question of what should the woman in the picture do when a man is trying to touch her private parts by saying:

‘I would tell her if you are both ready then fine but if she is not ready, he must respect her.’ (Ella, Female, 45, English, FG5)

‘Only if a woman gives permission to a man, then they can go to bed together like that.’ (Thato, Female, 41, Afrikaans, FG3)

The participants in both the male and female groups could identify same sex relationships, for example, when shown pictures or cut-out characters depicting such. Some participants would point at a picture and say ‘lesbians’ or ‘gay couple’ with an obvious discomfort shown by their facial expressions or body language. There was heteronormative dominance in all the groups and general avoidance of responding to prompts about relationships between people of the same sex. When given the opportunity to match various cut-out characters who could be interested in a sexual relationship, none of the participants matched same sex relationships. When the facilitators matched a potential same sex relationship, some male participants showed hostility and strong disapproval, for example, one insisted:

‘Male and male, no! Female and female, no! Only male and female.’ (Joe, Male, 28, Afrikaans, FG4)

For many, television (TV) seemed the most familiar reference to understanding same sex relationships rather than their own lives or community. For example, one female participant said:

‘Yes, I saw it on TV. Two lesbians and they live in a house together and they were kissing one another and one gave a ring to the other.’ (Leah, Female, English, 39, FG2)

One of the male participants said the following about men having sexual relations with other men; which he related to a British gay singer who died of AIDS in the 1990s:

‘I know about it because Freddie Mercury died of it. I should not do it, it’s wrong.’ (Victor, Male, 47, English, FG6)

### Knowledge and awareness of sexual violence

This theme comprises two sub-themes: *Identification and reacting to risk* and *Knowledge of terminology for sexual violence.*

#### Identification and reacting to risk

Like the others, this theme showed that the participants had a degree of knowledge of some risks, but they also struggled to identify others. They showed knowledge of inappropriate touch which they referred to as ‘dangerous’:

‘Now it’s becoming dangerous. That part that should not be touched. On the wrong spot…totally red light.’ (Prisca, Female, 36, Afrikaans, FG1)

‘Yes…underneath the bra…and underneath the panty parts…the guys touch the ladies…whole naked…and then they start getting pregnant…it’s dangerous.’ (Talent, Female, 39, Afrikaans, FG3)

They were also aware that if an older person had sex with a child, it would be abuse through recognising too wide an age gap between characters:

‘I think that one will be too young for him. He’s a parent. He must actually go with this one.’ (Mavis, Female, 28, English, FG5)

They showed the understanding that a perpetrator can be known to the abused person:

‘I think someone in the family…or perhaps an uncle…or a stranger….’ (Mavis, Female, 28, English, FG5)

They struggled with identifying lures either from a person known to them or someone they have just met. Some thought that they could agree to going to the person’s house but say no to sex, not realising that they put themselves into a compromising position by agreeing to go. For example, a social story was related about a girl who meets a man at church or at a social gathering. She finds him nice and he asks the girl to go to his house with her. With regard to this, some participants responded as follows:

‘She can go sometimes.’ (Lidia, Female, 32, Afrikaans, FG1)

‘She can go always.’ (Zoe, Female, 44, Afrikaans, FG3)

Some recognised the risk and did not agree with their peers:

‘She must know him well, then she will probably feel more comfortable. You can’t just go to anyone’s house that you don’t know.’ (Ella, Female, 45, English, FG5)

The risk of being lured on social media was not identified by most of them. One of the female participants thought it was enough to check if someone was in a relationship or married before accepting a friendship request on Facebook and she herself had confirmed a request from a stranger before:

‘Because for example I have someone I confirmed on Facebook and then he says, “what are you doing now?” then he said “can you send a picture of you?” and I said I don’t know you why do you want my picture? … because he sent an invite and I checked all those things like is he married or in a relationship… I wanted to chat.’ (Ella, Female, 45, English, FG5)

#### Knowledge of terminology for sexual violence

Most of them were not familiar with the words ‘rape’ or ‘sexual abuse’. Upon being asked which words described what a picture or a story depicted, they said things like ‘It’s dangerous’, or ‘It can be called bullying’ or ‘bullying isn’t it’. They would eventually show that they have heard the words before when the researcher mentioned them which showed that they were not clear what exactly the words described. A few who were clear said:

‘He’s touching her private parts, forcing himself on her and it’s called rape.’(Victor, Male, 47, English, FG6)

‘Yes, it is rape, when his penis is forced into her private parts.’ (Betty, Female, 25, English, FG5)

The following were responses to a picture depicting inappropriate touch whereby a woman was trying to escape from a man who was touching her:

‘Force is a crime…and when he does it all the time, then you can go to the law…to tell them about it.’ (Deon, Female, 31, Afrikaans, FG3)

Another one responded to a picture of an older man touching a much younger boy’s private parts with the boy’s face showing that he was afraid and attempting to escape:

‘He’s touching…That’s what we call touching.’ (Luke, Male, 29, IsiXhosa, FG6)

### Past experience of abuse and/or attempted abuse

Five women from three of the women’s groups shared that they had either experienced forced sexual intercourse, attempted forced sexual intercourse or forced touch before. They were not asked for this information, but voluntarily shared it. Thus, it is not clear if there were other abuse survivors who decided not to reveal such information. None of the male participants disclosed sexual abuse. Most of the women shared this information in response to the questions on what the character should do when faced with a risk of abuse or after being abused. While talking about the right to say no, one participant shared how she resisted unwanted touch from a male bus driver and went on to report the incident. This was an important teaching moment, whereby the researchers affirmed the young woman’s courage in reporting and seeking help. Such moments also opened the conversation to hear others’ thoughts and allowed the researchers to further emphasise the right of not to be abused, to seek help when needed and to report. She said:

‘Once I also went through that, when the guy that drove the school bus touched me…He touched me here (showing her groin). I told him no, but he didn’t want to listen… He said I must keep quiet but I did not. I did and he lost his job.’ (Prisca, Female, 36, Afrikaans, FG1)

In response to the fact that one had shared her experience that she told and did not keep quiet, another participant said:

‘That’s what the uncle said to me…but I spoke out. Some of the kids are forever staying quiet…and the men in turn will just carry on… I spoke…I didn’t listen to him who said that we should keep this secret. I spoke.’ (Lidia, Female, 32, Afrikaans, FG1)

Another one shared what happened after she had been raped. She shared about how she went through counselling with a psychologist. She was more comfortable with opening up to the psychologist than her own mother:

‘Apologies…when it happened to me, I saw a psychologist…and spoke about my experience… I spoke openly to the psychologist…because I was too scared to speak to my mother…because stories have a life of their own, my mother can tell people.’ (Thato, Female, 41, Afrikaans, FG3)

One of the participants shared about a traumatic experience of being raped while travelling on the train. It was evident that although the case had been reported and she had been through counselling, she was still suffering from the trauma as she avoided looking at pictures showing sexual behaviour. She was given the option to withdraw and go to speak to a counsellor, but she said she wanted to continue and she did not need any more counselling. Her response to the pictures was:

‘I don’t really like it that touching. I don’t like it. I don’t like to look at the picture (looking away with a frowning face)… I don’t like these men. These men are not right. The man hurt me. He gave me AIDS… A man is a devil…and I showed the police that’s the guy who did it to me. On my body…in the train…He tore at me…tore at me…and tore my pants… He told me that I must go with him…and he was going to pay me ten rand.’ (Mary, Female, 37, Afrikaans, FG2)

### Knowledge of actions to take in case of abuse

This theme showed a distinction between the level of knowledge shared by those who revealed past experiences of abuse and those who did not. They expressed the need to report to police, a family member or social worker and to go for a medical check-up. The police were identified as the most common place to go to report the incident of abuse and social workers were also identified as important role players:

‘Yeah, to go to the police station so that the police can come and arrest the guy so that he gets set for the rest of his life to go to jail and so that it can be talked out in court.’ (Betty, Female, 25, English, FG5)

‘It might happen again and it might be worse so she needs to tell. Maybe if she has a social worker. It depends where she works… (laughing).’ (Thato, Female, 41, Afrikaans, FG3)

On the contrary, there were some with erroneous knowledge and perceptions of whether to tell or not if one was abused. For example, in three of the four women’s groups there were participants who said that it was not necessary to report if the abuse only happened once and one should only report if it recurs:

‘If the man does it for the first time, you can keep it to yourself but if he keeps doing it you must report. Only tell if he does it again.’ (Deon, Female, 31, Afrikaans, FG3)

There was also the belief that the victim of abuse can choose whether to report or not:

‘Go straight to the police, and report what happened to her. On the other hand, if she refuses to report immediately…it’s up to her as she’s keeping it to herself.’ (Betty, Female, 25, English, FG5)

The impact of the incident on one’s emotional and mental health were also identified:

‘Maybe he talk it out, maybe to his family… If he wants to, if he doesn’t want to he can leave it. But I also think things like that you cannot let it stay inside because it’s bothering you, it won’t be good for you.’ (Dylan, Male, 44, Afrikaans, FG4)

Some, from their past experiences, were clear of the need for preserving evidence for forensic examination and to have a medical check-up:

‘No, she doesn’t need to wash because that is evidence. Otherwise the police can’t see…ok this lady was raped…or this lady wasn’t raped. If she washes her, then they can’t see anything … but if she doesn’t wash her … and then they will see.’ (Molly, Female, 39, English, FG2)

Some expressed lack of confidence and fear of being blamed as deterrence to reporting:

‘Yes, the mom can shout at her and they can have an argument. She can change the story because she is too scared to tell the truth.’ (Ella, Female, 45, English, FG5)

‘Maybe he will think his parents will be like ‘why did you let the other person touch you in the first place?’ (Victor, Male, 47, English, FG6)

## Discussion

The study attempted to explore adults with intellectual disabilities’ experiences, perceptions and knowledge of risks to sexual violence. Their responses revealed that both men and women had some knowledge of risks to sexual violence, but they also had knowledge gaps and some erroneous knowledge and perceptions that could further increase their risk of sexual abuse and decrease their ability to seek help to stop sexual abuse if it occurred. The sources of their knowledge were a subject beyond the scope of the study, but some of their statements implied that those supporting them like family caregivers and service providers were their sexuality educators. Many of them had experiences of love relationships which were mostly described as non-sexual. Only female participants voluntarily disclosed the experiences of abuse and there was a direct correlation between the actual experience of abuse and knowledge of risk and awareness of the appropriate actions to take in case of being a survivor of attempted or actual sexual abuse. This section presents implications of the findings and suggests directions for future research, practice and policy framework.

The perceptions and experiences of love relationships were deemed critical in this study, because understanding of healthy, consenting sexual relationships is important for one to be able to recognise abusive relationships. The fact that most of the women participants expressed a preference to be in non-sexual relationships makes it difficult to know whether they would have the agency to distinguish between a safe, consensual sexual relationship and an abusive relationship. A preference for non-sexual relationships could be reflecting what Bernet and Ogletree (2013) found in their study that women with intellectual disabilities chose abstinence because of fear, previous negative sexual experiences, just avoiding coping with sex itself or the idea of engaging in sex. Negative messages about sex from parents and professionals could also contribute to their choices as implied by some of the narratives. A previous study in the same setting found that parents preferred their young adults with intellectual disabilities to form friendships for companionship, but they were against intimate or sexual relationships (Kahonde 2016). The fact that the parents had to assent to the adults’ participation in the current study could have favoured the participation of those whose parents accentuated the abstinence-only discourse.

In another South African setting, educators prioritised protection and the risk discourse and avoided teaching about sexual activity (Hanass-Hancock et al. 2018). In the current study, the findings are illustrative of this ‘no touch’ emphasis by both parents and service providers. Hence, people with intellectual disabilities need education, not only to protect them from sexual violence but also to develop sexual agency as emphasised by Hanass-Hancock et al. (2018) and to understand their rights in a relationship.

All the participants could recognise inappropriate touch and they could also recognise other forms of sexual abuse including rape as depicted by the pictures and narrated in the social stories. On the other hand, they equally struggled to identify some lures which could make them comply with the requests of the abuser. Their responses corroborate the findings that people with intellectual disabilities may comply with the request of others without realising potential dangers (Barger et al. 2009; Smeaton & Franklin 2018), even though some of our participants were clear about the dangers. Within South Africa, where risk and gender-based violence is exceptionally high, people with intellectual disabilities need constant messages about the dangers of being lured to follow people to secluded places even if they know them. We also suggest that each person with intellectual disability is supported to know and name at least two trusted people whom they can speak to if and when they need help. Needless to say, sexual violence prevention programmes for people with intellectual disabilities in South Africa need to take a comprehensive approach while also emphasising teaching correct terminology as the vague terminology used by some of the adults could be a barrier to reporting the cases of abuse.

A review by Barger et al. (2009) on international literature on the programmes for sexual assault prevention targeting women with intellectual disabilities found four programmes, two from Australia and two from the USA. They recommended comprehensive programmes that emphasise the awareness of risk, assertiveness and self-confidence while including all the stakeholders in the lives of people with intellectual disabilities like family, formal carers, professionals, friends and having people with intellectual disabilities involved in the development, implementation and evaluation processes. Following the findings of our study, we agree with Barger and colleagues, and recommend the development of such programmes in South Africa by services working on sexuality education programmes that communicate about healthy and unhealthy relationships and increase the awareness of the needs of abuse survivors in their recovery and healing. Such programmes should be gender sensitive and disability inclusive.

There is currently a lack of focus on identifying the needs of men with intellectual disabilities as research is biased towards sexual violence against women (Barger et al. 2009; Bernet & Olgetree 2013; Bornman & Rathbone 2016; Haffejee & Theron 2017). The obvious reason given in the studies is that women are more at risk which is evidenced by the statistics of female victims both within the population of people with disabilities (Broban et al. 2020) and among people with intellectual disabilities. Although we did not aim for an in-depth gendered analysis in this article, we recognised the differences in the experiences of the women and men, for example, women tended to share experiences of past abuse and they suggested more passive ways of reacting to abuse. These findings call for support and empowerment interventions that cater for the specific needs of men and women. Furthermore, it is imperative to explore how dominant heteronormative perceptions affect relationship beliefs and self-efficacy of women and men with intellectual disability. For example, the hostility expressed by some male participants towards a potential same sex relationship could make it more difficult for them to access information or support about same sex sexual behaviour, recognise same sex abuse and/or increase their reluctance to seek help if they have experienced same sex abuse. Also, the tendency to associate same sex relationships with what is seen on television could be an indication of ignorance which may further exacerbate reluctance, denial, and hostility towards same sex relationships.

The number of female participants who shared their experiences of sexual violence or attempted sexual violence confirm the alarmingly high rates of abuse among women with intellectual disabilities. Not only strangers but people responsible for the care and support of these women like school bus drivers and uncles were implicated and it is apparent that the women are at risk in public places like trains, buses and at church. These findings are in line with the literature of abuse of women in general in South Africa, whereby rape can occur in public places like post offices or schools (Lyster 2019; Nyokangi & Phasha 2016). A study conducted in the Western Cape Province by Dickman and Roux (2005) described 100 cases of sexual abuse assessed by the Sexual Assault Victims Empowerment Programme between 1990 and 2000 comprising 94 complainants. They reported that in 89% of the cases, the perpetrator was known to the complainant, with 23% having familial connections with the complainant and three being staff members. The occurrence of abuse perpetrated by people who are known or family members present challenges when designing feasible prevention interventions as argued by Barger et al. (2009) and providing support to the abuse survivor. For example, a person with intellectual disability may lack support in accessing intervention programmes if the perpetrator is a close family member or primary caregiver and the abuse may be minimised or denied by the family. More conversations need to happen with people with intellectual disabilities to encourage them to speak up, to ensure early identification of those at risk and bring the perpetrators to book. Every organisation should have abuse prevention awareness education sessions and outreach. This needs to be supported by increasing family education around abuse and making them aware of the law and their responsibility to report abuse as well as training in the justice and police system to accommodate the needs of people with intellectual disabilities. Furthermore, policy reform is imperative in South Africa to enforce strict screening of the staff members working with people with intellectual disabilities, preclude employment of sex offenders and make the perpetrators serve heavier sentences rather than simply losing one’s job, as in the case shared by one of our participants.

The participants were familiar with the need to report and seek help in case of abuse, but there were also some who were either unsure or lacked confidence to report abuse. A matter of concern was the common erroneous idea among some participants that if an abuse happens only once then it is not a crime but one must report if it happens repeatedly. This can put them at risk of accepting unwanted sexual lures from perpetrators as long as it is the first time or not reporting it if it only happens once. Further exploration of the sources of such beliefs and understanding of abuse is needed in the research context. Furthermore, sexuality education and abuse prevention interventions for people with intellectual disabilities should emphasise that abuse is a crime even if it happens once. No previous literature was found reporting such findings and we argue that researching sexual abuse through exploring the perceptions and experiences of people with intellectual disabilities reveals critical concepts that cannot be known when relying on other people’s subjective narratives.

Knowledge of people with intellectual disabilities’ individual circumstances is important as some may not be believed by their families or caregivers, or they may not trust their families, or they may withhold information out of fear. Studies from other settings in South Africa revealed that socio-cultural conceptions of intellectual disability may also influence the family and community responses to sexual victimisation of people with intellectual disabilities which have been elucidated earlier (Hanass-Hancock et al. 2018; Meer & Combrinck 2015). Hence, with Phasha (2009), we argue that sexual abuse is a community problem which needs to be addressed through community-oriented interventions. In line with this, Curtiss and Kammes (2020) highlighted the needed to use an ecological framework based on the Ecological Systems Theory (Bronfenbrenner 1979) to address sexual violence against people with intellectual disabilities. Curtiss and Kammes (2020) contend that sexuality education on its own is insufficient to prevent sexual violence but there is need to address vulnerabilities at each level of the system from individual, family, work, school, community, cultural institutions, social structures, policies and the broader cultural context. Having suggested multi-sector interventions to prevent sexual violence against people with intellectual disabilities in the setting for the current study, an ecological framework is likely to be effective as a lens to approach these interventions. The ecological framework will enable a comprehensive approach, as suggested not only by the current study but also previous studies in South Africa that reported the individual factors influencing the risk to sexual violence against people with intellectual disabilities as well as the perceptions, beliefs and practices of families, communities, and professionals working with this group (Meer & Combrinck 2015; Phasha & Myaka 2014). Additionally, an ecological framework will enable a shift from only looking at people with intellectual disabilities as an inherently vulnerable group that needs protection to addressing environmental factors like societal ableism and rape culture.

## Conclusions

The study was an initial step in exploring the knowledge, perceptions and experiences of sexual violence against people with intellectual disabilities in Cape Town. Involving adults with intellectual disabilities enabled the researchers to bring out the nuances of their perceptions and experiences which are critical for developing and implementing programmes to empower and protect them from sexual violence. Researching the subject of sexual violence through a series of focus groups was beneficial for the participants as they learnt from each other and were also encouraged to share their own perceptions and stories in a space that was safe. We also identified the potential for peer support and peer education. The participants who were more knowledgeable of risks and with confidence to share their experiences inspired others who seemed to lack confidence to speak up in the group sessions. On the other hand, the experiences of others were important as reference points for the researchers to educate other group members.

This study raised the need for proactive interventions and support to prevent sexual violence against people with intellectual disabilities as the adults seem to be learning through reactive support given after an incident of sexual violence. Based on our findings, we suggested ways by which the existing gaps in supporting people with intellectual disabilities through prevention of sexual violence can be addressed. We conclude by reiterating the need for more research involving people with intellectual disabilities themselves and the different stakeholders in their lives so as to develop context and gender-specific theoretical frameworks to explain sexual violence against people with intellectual disabilities. To achieve this, we suggested the relevance of an ecological framework as a guiding tool in both research processes and implementation of the outcomes.

## Limitations

The study was not without its own limitations. Firstly, the findings are based on verbal responses from participants without observation of their possible responses to different exemplary situations of abuse. It is not clear whether the knowledge they have, including what they were taught during focus group sessions, will be translated into action if they are faced with risks. Secondly, their communication abilities were diverse and those with more verbal communication dominated the discussions although the researchers were alert to this and kept encouraging everyone to respond. Thirdly, we did not investigate the participants’ knowledge of other factors that could increase risks like substance abuse. Fourthly, despite the advantages of focus group discussions stated earlier, the group setting of a focus group may have inhibited some participants from expressing themselves fully. For example, heteronormative concept which prevailed in each group possibly inhibited voluntary discussion on same sex relationships. Lastly, we recognise the possible limitations of having staff members as supporters during interviews, especially when discussing sensitive subjects like sexual abuse, for which the staff members have to intervene often. On the other hand, we deemed it as a strength of our data collection process as the staff members knew the participants well and understood their communication styles which was helpful in facilitating and supporting communication between the researchers and participants whenever needed. We suggest that this is an area that needs further attention in research involving adults with intellectual disabilities. However, this study plays a critical role in being the first of its kind to initiate the needed conversations and responses to the scourge of sexual violence against people with intellectual disabilities in the study setting despite these limitations.
